# Effects of unilateral airway occlusion on rib motion and inspiratory intercostal activity in dogs

**DOI:** 10.14814/phy2.13242

**Published:** 2017-04-10

**Authors:** Dimitri Leduc, Sarah Marechal, Olivier Taton, Bernard Blairon, Alexandre Legrand

**Affiliations:** ^1^ Laboratory of Cardiorespiratory PhysiologyBrussels School of Medicine and Chest ServiceErasme University HospitalBrusselsBelgium; ^2^ Laboratory of PhysiologyMons School of MedicineMonsBelgium

**Keywords:** Intercostal muscles, mechanics of breathing, pleural pressure transmission, respiratory muscles, rib cage motion

## Abstract

Unilateral bronchial occlusion, a complication of many lung diseases, causes dyspnea but the mechanism of this symptom is uncertain. In this study, electromyographic (EMG) activity in the parasternal and external intercostal muscles in the third intercostal space and inspiratory motion of the third rib on both sides of the thorax were assessed during occlusion of a main bronchus for a single breath in anesthetized dogs. Occlusion produced a 65% increase in external intercostal EMG activity in both hemithoraces without altering parasternal EMG activity. Concomitantly, the inspiratory cranial rib motion showed a 50% decrease on both sides of the thorax. These changes were unaffected by bilateral vagotomy. However, when an external, caudally oriented force was applied to the third rib on the right or left side so that its inspiratory cranial displacement was abolished, activity in the adjacent external intercostals showed a twofold increase, but rib motion and external activity in the contralateral hemithorax remained unchanged. It is concluded that during occlusion of a main bronchus, the increase in external intercostal activity is induced by the decrease in inspiratory cranial rib displacement in both hemithoraces, and that this decrease is determined by the increase in pleural pressure swings on both sides of the mediastinum. This mechanism, combined with the decrease in PaO_2_, induces similar alterations when unilateral bronchial occlusion is maintained for a series of consecutive breaths.

## Introduction

Occlusion of a main bronchus is a common complication of bronchial cancer but can also occur in nonneoplastic pathologies, such as after inhalation of a foreign body or after accumulation of bronchial secretions. This setting is frequently associated with dyspnea (Scoggin 1988), but the mechanism of this symptom remains unclear.

It would be expected that occlusion of a main bronchus would elicit a number of alterations. First, based on the previous observations of tracheal occlusion in dogs (De Troyer 1991b), it would be expected that occlusion of a main bronchus would impede the cranial motion of the ribs, the caudal motion of the diaphragm, and the shortening of the inspiratory muscles, including the diaphragm and the parasternal intercostal muscles, in the ipsilateral hemithorax (De Troyer 1991b). Based on the length‐tension relationship of these muscles, it would therefore be expected that, during inspiration, the pleural pressure swing (ΔPpl) would increase on the occluded side (Farkas et al. 1985; Road et al. 1986; Boriek et al. 2006). Second, the decrease in the cranial motion of the ribs should also increase the inspiratory EMG activity in the external intercostal muscles on the occluded side (De Troyer 1992, 1996). However, De Troyer demonstrated (De Troyer 1991a) in dogs that the ΔPpl generated during unilateral phrenic nerve stimulation or that generated during unilateral contraction of the inspiratory intercostal muscles is, to a large extent, transmitted through the mediastinum to the contralateral hemithorax. Consequently, it would be expected that occlusion of a main bronchus would induce an increase in ΔPpl in both hemithoraces and that this would result in a decrease in the cranial displacement of the ribs and an increase in external intercostal muscle activity on both sides of the rib cage (De Troyer 1996).

The present studies were specifically designed to test these predictions. Firstly, we assessed inspiratory muscle activity and rib cage motion on both sides of the rib cage in anesthetized dogs while the right or the left main bronchus was occluded for a single respiratory cycle. We then measured muscle activity and rib motion when bronchial occlusion was maintained for a number of breaths.

## Methods

The studies were carried out on eight adult bred‐for‐research dogs (20–24 kg body wt). Studies were approved by the Animals Ethics and Welfare Committee of the Brussels School of Medicine. The animals were anesthetized with pentobarbital sodium (initial dose 30 mg/kg iv), placed in supine posture, intubated with a cuffed endotracheal tube, and connected to a mechanical ventilator (Harvard Pump, Chicago, IL). A venous canula was inserted into the forelimb to give maintenance doses of anesthetic (3–5 mg/kg per h), and a catheter was placed in a femoral artery to monitor blood pressure and heart rate and to sample blood for blood gasses analysis (Roche Cobas B 123 POC system ‐ Switzerland). The rib cage and intercostal muscles were exposed on both sides of the chest from the first to the ninth rib by reflection of the skin and the superficial muscle layers. A tracheostomy was then performed through a midline incision of the neck and the vagi were bilaterally isolated, after which two endotracheal tubes (*n*° 5–7) were positioned in the right and left main stem bronchi. These tubes were positioned under endoscopic guidance to ensure patency of all lobar bronchi, and they were tethered to the tracheal rings above and below the site of tracheostomy so as to avoid any subsequent inadvertent displacement. A differential pressure transducer (Validyne Corp., Northridge, CA) was connected to a side port of each endobronchial tube to measure the changes in airway opening pressure (ΔPao). Electromyographic activity (EMG) of the parasternal and external intercostal muscles in both hemithoraces were recorded using pairs of silver hook electrodes spaced 3–4 mm apart. Each electrode pair was placed in parallel fibers and inserted into the muscle area known to receive the greatest neural inspiratory drive. Therefore, the parasternal intercostal electrodes were implanted in the third interspace in the muscle bundles near the sternum (De Troyer et al. 2005), while the external intercostal electrodes were positioned in the dorsal portion of the third interspace, immediately ventral to the rib angle (Kirkwood et al. 1982; Greer and Martin 1990; De Troyer et al. 2005). The four EMG signals were processed with amplifiers (model 830/1; CWE; Ardmore, PA), band‐pass filtered below 100 and above 2,000 Hz, and rectified before their passage through leaky integrators with a time constant of 0.2 sec.

A hook was then screwed bilaterally into the third rib in the midaxillary line and connected to a linear displacement transducer (Schaevitz Engineering, Pennsauken, NJ) in order to measure the craniocaudal (axial) rib displacement (Xr), as described previously (De Troyer and Kelly 1982).

The animals were given 30 min recovery time after instrumentation. Following this, ΔPao's, EMG activities of the external and parasternal intercostal muscles, and the craniocaudal motion of the third rib of both sides of the rib cage were recorded. The experiment was performed in three stages.

In the first stage, every 10–15 breaths, one of the endotracheal tubes was occluded at resting end‐expiration for a single inspiratory effort. Ten occluded breaths were recorded for each side of occlusion in each animal.

In the second stage, the procedure was repeated but the unilateral bronchial occlusion was sustained for 10 consecutive breaths. Blood gas analysis was performed before and after the 10th occluded breath.

As it was observed that unilateral airway occlusion induced a bilateral EMG increase in external intercostal muscles, we would assess whether a segmental reflex could be transmitted through the midline in the spinal cord to contralateral motoneurones. Therefore, in the final stage, while the animals were spontaneously breathing and both endobronchial tubes were open, an external caudally oriented force was applied at intervals to the right or the left third rib to suppress the rib cranial displacement during inspiration. This procedure was performed in five of eight animals.

After these procedures were completed, in each animal, the vagi nerves were infiltrated with 2% lidocaine (lignocaine) and sectioned. The first two stages of the experiment were then repeated.

The animals were maintained at a constant level of anesthesia throughout the experiment. The anesthesia was regulated so that the animals' blink and corneal reflexes were active throughout the measurements. Rectal temperature was also kept constant between 36 and 38°C with infrared lamps. At the end of the experiment, the animals were given an overdose of anesthetic (30–40 mg/kg iv).

### Data analysis

In the first stage of the experiment, the inspiratory axial displacement of the ribs and phasic inspiratory EMG activity in external intercostal and parasternal intercostal muscles on both sides of the rib cage were averaged over 10 consecutive occluded and the 10 preceding unimpeded breaths. Axial rib displacement (Xr) was expressed in absolute value in mm. Inspiratory EMG activity was first quantified by measuring the peak height of the integrated EMG signal in arbitrary units, and was then expressed as a percentage of the activity recorded during the inspiratory cycle just before the occluded breath. In addition, inspiratory time (Ti) was measured from the onset of the parasternal intercostal electromyogram until peak activity, and the peak deflection of the integrated EMG signal was also divided by Ti to obtain the average rate of rise of inspiratory activity.

In the second stage of the experiment, inspiratory EMG activity in external and parasternal intercostal muscles for each respective occluded respiratory cycle was expressed as a percentage of the EMG inspiratory activity recorded during the unimpeded cycle just before occlusion.

In the final stage, inspiratory EMG activity was also expressed as a percentage of the EMG inspiratory activity recorded during the cycle preceding the rib manipulation.

In all three stages of the experiments, results were not significantly different whether bronchial occlusion or suppression of rib motion was performed on the left or right side. Therefore, results were pooled and expressed as ipsi‐ or contralateral relative to the side of bronchial occlusion or rib manipulation.

The data were finally averaged over the animal group and presented as means ± SE. Paired t test was used for statistical analysis of the effects of isolated unilateral bronchial occlusion or rib manipulation. ANOVA with repeated measures was used for the statistical analysis of the effects of sustained unilateral bronchial occlusion, and Fischer test of LSD type (Least Significant Difference) was used for multiple comparison testing of the mean values, as appropriate. The criterion for statistical significance was taken as *P *<* *0.05.

## Results

### Effect of unilateral bronchial occlusion during a single inspiratory cycle

**Figure 1:**
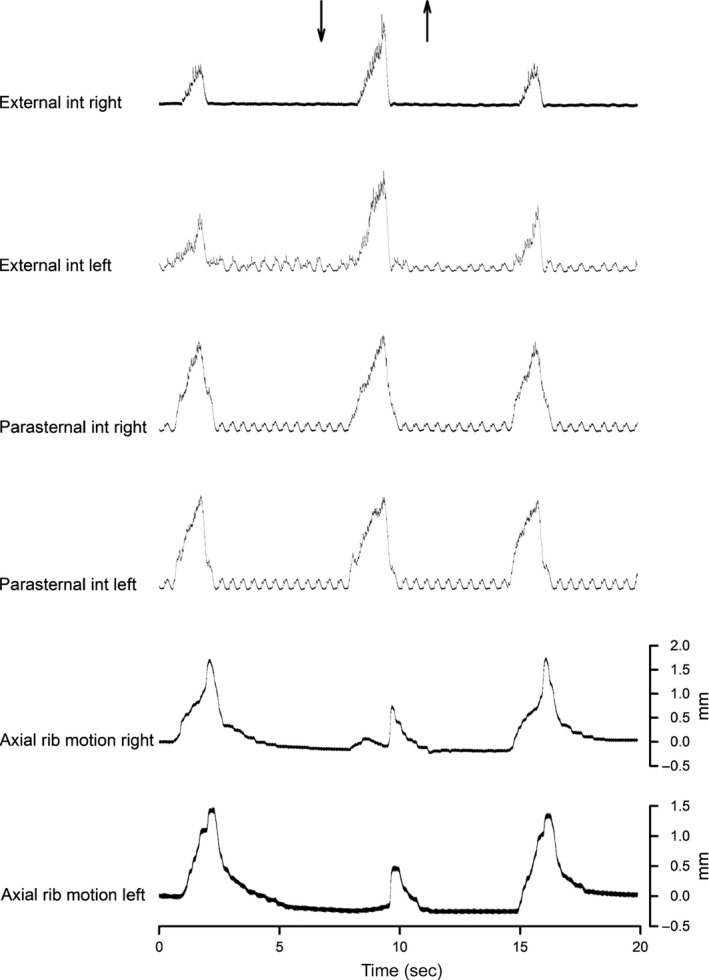
Traces of parasternal and external intercostal EMG activity (integrated signals) and axial displacement of the third rib (cranial displacement upward) on both sides of the chest obtained from a representative animal during resting room air breathing in the control condition and during occlusion of the right main bronchus for a single breath (arrows). Note the marked increase in EMG activity in external intercostal muscles and the decrease in cranial motion of the ribs. EMG activity in the parasternal intercostal muscles, however, remained unchanged.

**Figure 2:**
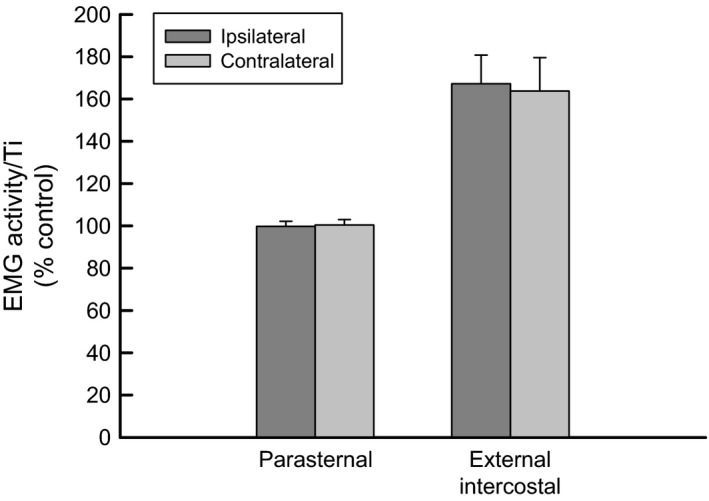
Parasternal intercostal and external intercostal inspiratory EMG activity on both sides of the thorax during unilateral bronchial occlusion for a single breath. EMG/Ti activities are expressed as percentages of activity recorded during control. Note the marked increase in external intercostal activity on both sides of the thorax during occlusion. Values are means ± SE from the eight animals.

The records of external intercostal and parasternal intercostal EMG activity in the third interspace and of the inspiratory motion of the third rib on both sides of the rib cage obtained in a representative animal during control breathing and during unilateral bronchial occlusion are shown in Figure 1. The mean ± SE values of EMG obtained in the eight animals are displayed in Figure 2. ΔPao developed in the occluded bronchus was −10.2 ± 2.5 cm H_2_O.

During unilateral bronchial occlusion, the inspiratory EMG activity recorded from the third parasternal intercostal muscle remained unchanged, both when occlusion was performed on the ipsilateral side and on the contralateral side. EMG/Ti, expressed as a percentage of control were, respectively, 99.8 ± 2.4% (*P* = 0,869) and 100.5 ± 2.6% (*P* = 0.805). It is worth noting that peak EMG activity in the parasternal intercostal increased slightly due to an increase in Ti during occlusion (1.17 ± 0.28 sec during occlusion vs. 1.02 ± 0.21 sec before occlusion, *P* < 0.001). In contrast, the inspiratory EMG activity recorded from the third external intercostal muscle on both sides of the rib cage was significantly increased during unilateral bronchial occlusion. EMG/Ti of the external intercostal muscle on the ipsilateral side reached 167.2 ± 13.6% of control (*P* < 0.001), and EMG/Ti of the external intercostal muscle on the contralateral side was 163.8 ± 15.7% of control (*P* < 0.001). This increase in external intercostal EMG activity was similar for both sides of the rib cage (*P* = 0.819).

**Figure 3:**
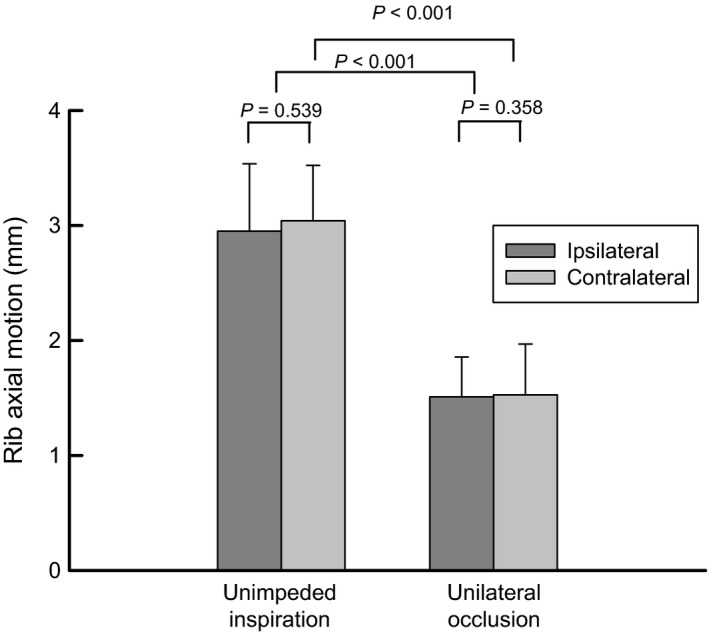
Inspiratory motion of the third rib on both sides of the thorax during unimpeded inspiration and during unilateral bronchial occlusion. Note the marked decrease in the inspiratory cranial motion of the rib on both sides of the thorax during occlusion. Values are means ± SE from eight animals.

The impact of unilateral bronchial occlusion on inspiratory rib motion is shown in Figure 3. During occlusion, the cranial inspiratory motion of the third rib was significantly decreased on both sides of the rib cage. It was 2.95 ± 0.59 mm before occlusion and 1.51 ± 0.35 mm during occlusion (*P* < 0.001) on the ipsilateral side of the rib cage, and 3.04 ± 0.48 mm before occlusion and 1.53 ± 0.44 mm during occlusion (*P* < 0.001) on the contralateral side. The magnitude of the decrease was similar for both sides of the rib cage.

### Effect of sustained unilateral bronchial occlusion

**Figure 4:**
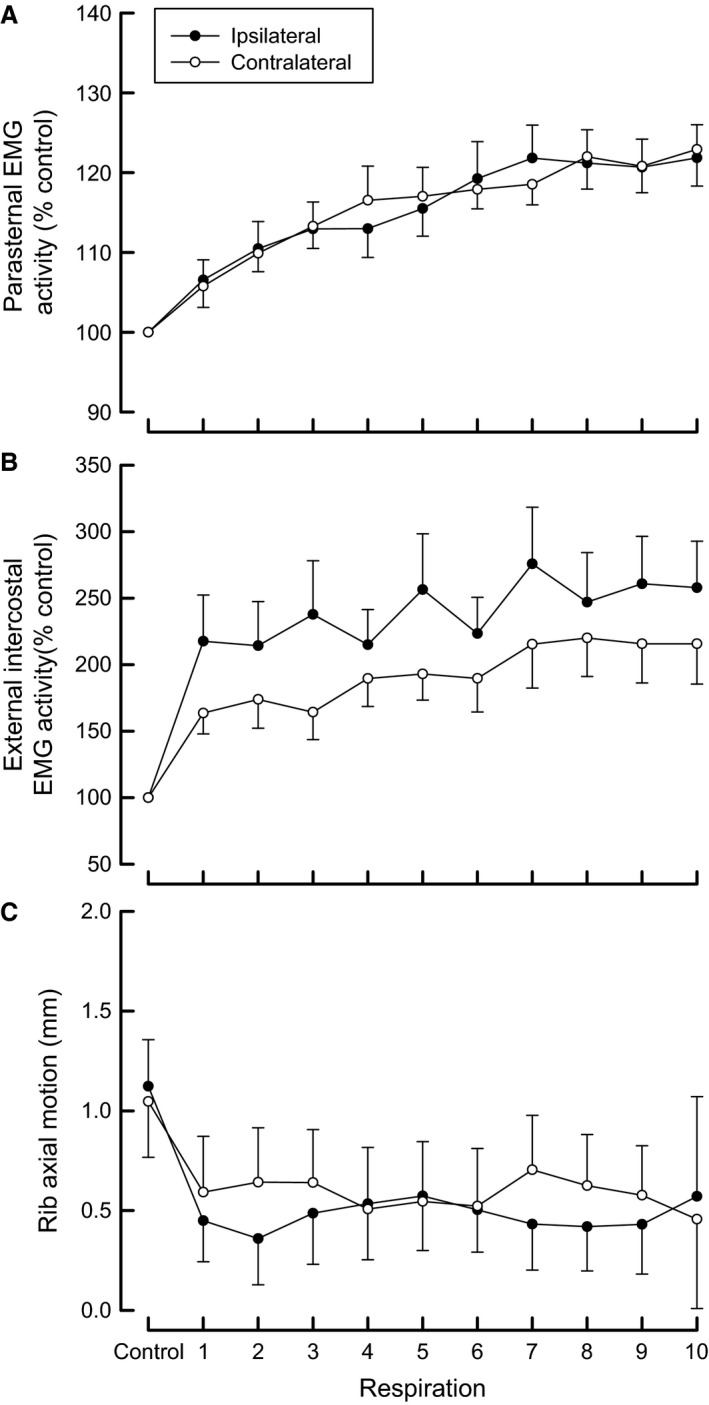
Parasternal intercostal and external intercostal EMG activity, and inspiratory cranial rib displacement on both sides of the thorax during unilateral bronchial occlusion sustained during 10 respiratory cycles. EMG activities are expressed as percentages of control activity. Note the progressive increase in EMG activity in the parasternal and external intercostal muscles on both sides of the thorax. Inspiratory rib motion decreased in the first occluded breath but remained unchanged during the subsequent occluded cycles. Values are means ± SE from eight animals.

The effects of sustained unilateral bronchial obstruction on parasternal intercostal and external intercostal EMG activity and rib displacement are shown in Figure 4 for the eight animals. During occlusion, the EMG activity of the parasternal intercostals gradually increased after the second respiratory cycle (*P* < 0.001). This increase in EMG activity was similar on the ipsilateral and contralateral side (*P* = 0.822), EMG/Ti reaching, respectively, 130.6 ± 3.5% of control and 132.8 ± 6.4% of control at the 10th occluded inspiratory cycle.

EMG activity in the external intercostal muscles also increased on both sides of the thorax, although this increase was significant in the first occluded cycle (*P* < 0.001 and *P* = 0.001, respectively, for ipsilateral and contralateral side). At the 10th occluded cycle, EMG/Ti for the external intercostal muscles reached 238.3 ± 33% and 220.8 ± 44.2% of the control value for ipsilateral and contralateral side, respectively.

The cranial inspiratory rib motion decreased during sustained occlusion and this was significant in the first respiratory cycle (*P* = 0.004 and *P* = 0.006, in both the ipsilateral and contralateral side). However, it remained unchanged afterward.

ΔPao in the occluded bronchus increased progressively from the first (−9.93 ± 2.93 cm H_2_O) to the 10th occluded inspiratory cycle (−12.69 ± 3.73 cm H_2_O, *P* = 0.036).

Blood gas analysis performed before occlusion and at the end of occlusion showed a decrease in PaO_2_ from 78.2 ± 13.1 mmHg to 58.4 ± 5.5 mmHg (*P* < 0.001). PaCO_2_, however, was not significantly altered (47.1 ± 7.6 mmHg and 48.2 ± 7.6 mmHg before and at the end of sustained occlusion, *P* = 0.06).

### Effect of vagotomy

Bilateral vagotomy did not alter the effect of unilateral bronchial occlusion during a single inspiration on EMG activity in the ipsilateral or contralateral parasternal intercostal (*P* = 0.853) and external intercostal muscles (*P* = 0.258). Cranial inspiratory rib motion increased after vagotomy (2.95 ± 059 mm during control vs. 3.39 ± 096 mm after vagotomy, *P* < 0.001). However, bronchial occlusion led to a decrease in ipsilateral and contralateral rib motion after vagotomy (1.88 ± 0.98 mm, *P* < 0.001).

Post vagotomy, sustained bronchial obstruction over 10 respiratory cycles continued to induce an increase in EMG activity in the parasternal intercostal and external intercostal muscles in the ipsilateral and contralateral side. Meanwhile, a decrease in the inspiratory motion of the third rib was recorded as before vagotomy.

### Effect of unilateral suppression of inspiratory rib motion

During unilateral retention of the third rib, the cranial inspiratory motion of the rib was abolished and EMG activity in the parasternal intercostal muscles remained unchanged (102 ± 3% and 102 ± 3% of control value and *P* = 0.21 and 0.13, respectively, in ipsilateral and contralateral muscle). In contrast, the EMG activity in the external intercostal muscle on the ipsilateral side of the rib cage was significantly increased, reaching 1940 ± 37.1% of the control value (*P* < 0.001), whereas on the contralateral side, external intercostal EMG activity did not change (97 ± 6% of control value, *P* = 0.28).

## Discussion

Previous studies in anesthetized dogs and rabbits (Corda et al. 1965; Sant'Ambrogio and Widdicombe 1965; De Troyer 1991b) have shown that bilateral airway occlusion for a single breath increases the inspiratory ΔPpl. This negative intrathoracic pressure outweighs the force exerted on the ribs by the inspiratory muscles of the rib cage and induces a decrease in the cranial motion of the ribs in the upper part of the rib cage. The resulting facilitation of external intercostal activity is referred to as the “load compensating” reflex (Corda et al. 1965; Sant'Ambrogio and Widdicombe 1965). It is induced by a stretch reflex acting on the muscle spindles of the external intercostal muscles and results in excitatory postsynaptic potentials in the corresponding *α*‐motoneurones. These potentials superimpose to the normal central respiratory drive and increase the efferent activity of *α*‐motoneurones to these muscles.

In addition, De Troyer (1992) and Romaniuk et al. (1992) demonstrated that, in contrast to the external intercostals, the EMG activity in the parasternal intercostals does not change during bilateral bronchial occlusion. This differing muscle response was attributed to the difference in the density of muscle spindles. In fact, in the dog, although the external intercostals contain large numbers of muscle spindles, the parasternal intercostals are poorly supplied (Duron et al. 1978).

Similarly, during unilateral bronchial occlusion in our animals, the cranial rib motion decreased in the ipsilateral hemithorax and a facilitation of EMG activity was observed in the ipsilateral external intercostal muscles but not in the adjacent parasternal intercostal muscles. It should be noted that, in fact, peak EMG activity increased during occlusion in parasternal intercostal as well as in external intercostal muscles. However, in the parasternal intercostal muscles, this increase was only related to the lengthening of Ti, whereas, in the external intercostal muscles, conversely to the parasternals, EMG/Ti was also increased signifying an EMG facilitation in this muscle.

One of the main findings of this study, however, was that unilateral bronchial occlusion induced similar increases in external intercostal EMG activity and similar decreases in the cranial inspiratory displacement of the rib cage on both sides of the thorax. A role of vagal afferent inputs in the alterations induced on contralateral hemithorax can be eliminated because these alterations persisted after bilateral vagotomy.

As discussed previously, unilateral bronchial occlusion leads to an increase in EMG activity in the ipsilateral external intercostal muscles through the activation of muscle spindles and the generation of excitatory postsynaptic potentials in ipsilateral *α*‐motoneurones. The possibility, therefore considered was that this segmental reflex would be transmitted through the midline in the spinal cord to the contralateral *α*‐motoneurones resulting in a bilateral EMG facilitation in external intercostals (Duron et al. 1978). We tested this hypothesis by eliminating the cranial inspiratory rib displacement on one side of the thorax during a respiratory cycle. Suppressing the cranial inspiratory movement of the rib elicited a large increase in inspiratory EMG activity in the ipsilateral external intercostal muscles but did not affect the contralateral external intercostal muscles, thus indicating that a segmental contralateral transmission of muscle spindle afferences could not be retained as the mechanism of this bilateral effect. Moreover, such a segmental contralateral transmission could not explain why the inspiratory rib elevation also decreased on the contralateral side of the thorax during unilateral bronchial occlusion.

**Figure 5:**
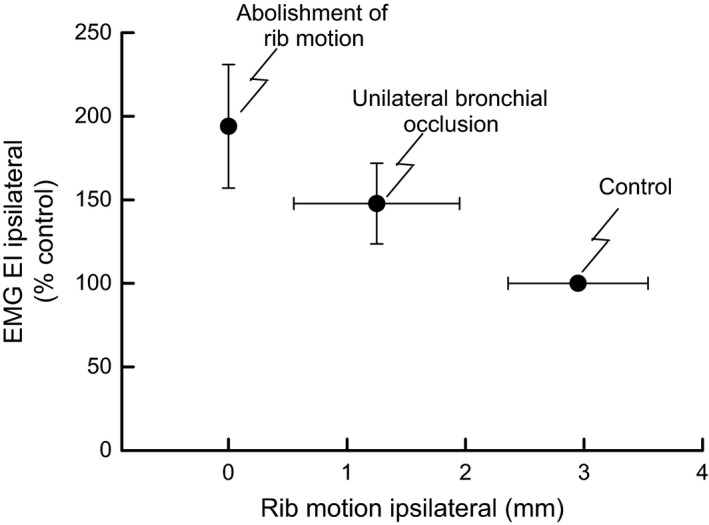
Relationship between the inspiratory axial motion of the rib and the peak EMG activity of the external intercostal muscle during control, unilateral bronchial occlusion, and during suppression of rib motion by an external force. Values are means ± SE from five animals.

The observation that unilateral bronchial occlusion produced a decrease in the inspiratory cranial displacement of the ribs and an increase in external intercostal activity on both sides of the rib cage, whereas a caudally oriented force on the ribs on one side of the thorax caused an increase in external intercostal activity only on the ipsilateral side, suggests that the increase in external intercostal activity during bronchial occlusion is closely related to the decrease in rib displacement. And indeed, previous studies using manipulation of rib motion in dogs (De Troyer 1996) and rabbits (Bonaert et al. 2012) have shown that when an external force is applied in the cranial direction to the ribs during inspiration, external intercostal activity decreases or disappears. Conversely, when the force on the ribs was applied in the caudal direction, external intercostal activity increased. Furthermore, the relationship between axial rib motion and external intercostal activity in this study was not only qualitative, but also quantitative, as shown in Figure 5. The suppression of cranial rib motion induced a twofold increase in external intercostal EMG activity, and unilateral bronchial occlusion induced a decrease in cranial rib motion, which reached approximately half of the control value, leading to a 50% increase in external intercostal EMG activity. These results are very close to those previously observed by De Troyer (1996).The relationship between axial rib motion and inspiratory EMG activity in the external intercostal muscles was attributed, at least in part, to the lengthening of the muscles which generates activation of muscle spindles.

On the basis of these considerations, the conclusion therefore emerges that unilateral bronchial occlusion caused an increase in ΔPpl on both sides of the thorax and, thus, that the greater ΔPpl in the ipsilateral hemithorax was transmitted through the mediastinum to the contralateral hemithorax. In agreement with the alterations observed during bilateral bronchial occlusion, the caudal motion of the diaphragm and the cranial displacement of the ribs in the ipsilateral hemithorax are impeded, leading to a lengthening of the diaphragm and parasternal intercostal muscle fibers. According to the active length‐tension relationship of these muscles (Farkas et al. 1985; Road et al. 1986; Boriek et al. 2006), the force developed by the diaphragm and the parasternal intercostal muscles is therefore increased, adding to the rise in ΔPpl in the ipsilateral hemithorax and this greater pressure loss is likely transmitted to the other side of the thorax. De Troyer (2008) has in fact shown that either unilateral phrenic stimulation or contraction of the inspiratory intercostal muscles on one side of the chest induces a fall of Ppl in both hemithoraces. Quantitatively, transmission to the contralateral hemithorax of the ΔPpl during hemidiaphragmatic contraction was approximatively 65% of the ΔPpl in the ipsilateral hemithorax, and transmission of the ΔPpl generated by unilateral inspiratory intercostal muscle contraction to the other side was 92% of that on the ipsilateral hemithorax (De Troyer 2008). It is therefore reasonable to believe that during unilateral bronchial occlusion, the pressure transmission from the ipsilateral to the contralateral side is the primary determinant of the reduction in inspiratory rib motion and the facilitation of external intercostal activity observed in both hemithoraces. Facilitation of external intercostal muscle activity during bronchial occlusion could also cause an increase in ΔPpl on both sides of the mediastinum, but, in view of the limited mechanical effectiveness of this muscle in anesthetized dogs (De Troyer 1991a), this contribution is likely to be small.

The mechanisms of compensation during unilateral bronchial occlusion were well illustrated when occlusion was maintained during a succession of breaths.

In agreement with the effect of single‐breath occlusion, EMG activity in the external intercostal muscle increased after the first occluded cycle and this EMG facilitation increased progressively during subsequent occluded cycles. EMG activity in the parasternal intercostal muscle did not change during the first occluded breath but it progressively increased after the third occluded cycle. This different outcome could be attributed to two concomitant mechanisms. The first is the facilitating segmental reflex mechanism described above which originates in the muscle spindles and elicites the immediate (first breath) increase in EMG activity in the external intercostal muscles. After three occluded respiratory cycles, EMG facilitation in parasternal intercostal muscles occurs. As the parasternal intercostal muscles in the dog are primarily governed by supraspinal command (De Troyer 1991b), the increase in parasternal EMG activity is most likely determined by an increase in central respiratory drive potentials.

These central drive potentials cause depolarization of the parasternals *α*‐motoneurones but they also increase depolarization of externals *α*‐motoneurones, leading to a progressive increase in EMG activity in both muscles.

The increase in central respiratory drive activity can be explained by hypoxemia, which we observed during sustained occlusion.

Finally, during sustained unilateral bronchial occlusion, we observed a bilateral decrease in the inspiratory cranial displacement of the ribs in the first occluded cycle. This rib displacement did not, however, decrease further during the following occluded cycles. This limitation of the effect on the rib displacement can likely be attributed to the concomitant increase in the force developed on the ribs by the parasternal intercostal and, for a less important part, by the external intercostal muscles. Apparently facilitation of EMG activity in these inspiratory muscles of the rib cage balances the effect of the progressive increase in ΔPpl on rib motion.

## Conflict of Interest

None declared.
